# OA06 Echoes of Hope: Navigating Pregnancy post heart transplant in the context of systemic sclerosis

**DOI:** 10.1093/rap/rkae117.006

**Published:** 2024-11-05

**Authors:** Aysha Gomaa, Brenda Stuart

**Affiliations:** University Hospitals Sussex, Brighton, United Kingdom; University Hospitals Sussex, Brighton, United Kingdom

## Abstract

**Introduction:**

Pregnancy in connective tissue disease presents unique challenges and considerations for both maternal and foetal health. These autoimmune disorders, characterized by inflammation can delay conception or complicate pregnancy with risks such as preeclampsia, preterm birth, foetal growth restriction and even foetal loss. Adding cardiac transplantation into the mix, matters can become even more complex. Optimal management requires a multidisciplinary approach, involving rheumatologists, obstetricians, and cardiologists, to carefully monitor and balance disease activity and medication safety. With vigilant care and individualized treatment plans, many women with connective tissue disease and organ transplant can achieve successful pregnancies and healthy outcomes.

**Case description:**

A 28-year-old female presented to Rheumatology at the age of 17 with a four-year history of Raynaud’s with digital ulceration, sclerodactyly and microstomia. Bloods showed a positive ANA with titre of 1:5120 and positive anti-Scl 70 antibodies. She was diagnosed with diffuse systemic sclerosis (SSc) and commenced on Mycophenolate Mofetil (MMF). As her disease progressed, she developed inflammatory cardiomyopathy, leading to heart failure, which necessitated heart transplantation at the age of 20. Post-transplant, she was on Tacrolimus, MMF and Prednisolone for immunosuppression. She currently has no signs of organ rejection, although a 2022 angiogram revealed mild atheromatous coronary artery disease. Her last echocardiogram in 2023 indicated good graft systolic function and right heart catheterization was unremarkable.

She has ongoing, though now less severe digital ulceration and necrosis secondary to Raynaud’s and vasculopathy. She was previously diagnosed with Kienbock’s disease and retinal vein thrombosis. Her treatment regime includes six-monthly Rituximab infusions, four-monthly Iloprost infusions and Nifedipine. Previously, she was on Sildenafil, but this was discontinued due to developing non-ischemic optic neuritis and retinal vein thrombosis.

Other complications secondary to SSc include oesophageal dysmotility, dyspepsia, and mild interstitial lung disease. Her medication history includes Bosentan, Losartan, Aspirin, Clopidogrel, Sertraline, Esomeprazole and multivitamins. She is currently stable and in disease remission with her current treatment regime.

The patient is eager to start a family and has attempted to conceive for nine months without success. Given her complex medical history, she has proactively sought comprehensive information to ensure a safe and successful pregnancy for both herself and the baby. MMF was switched to Azathioprine as part of her pregnancy planning. A joint cardiac-obstetric pre-pregnancy counselling session in 2022 reassured her that, despite the complexities, her medical conditions did not pose an absolute contraindication to pregnancy. She has now been referred to a fertility clinic.

**Discussion:**

Patients with autoimmune conditions and heart transplantation require lifelong immunosuppression. In this case, the combination of SSc and heart transplantation, along with mild atherosclerotic disease, increases the risk of pregnancy complications including pre-eclampsia, intrauterine growth restriction and preterm delivery.

Several factors need consideration when planning pregnancy, including the risk of organ rejection. With diligent monitoring of immunosuppressive medication, most solid organ transplants are unlikely to reject. Due to its teratogenicity, MMF should be switched to an alternative well in advance. Once patients are stable on a new immunosuppressive regimen, pregnancy can be contemplated. Other medications, such as Bosentan, should be stopped prior to pregnancy, and Losartan once pregnancy is confirmed.

Our patient may have a tendency towards vasculopathy given her extensive vasculopathy associate complications and there is a risk this can worsen during the pregnancy. However, a potential positive during the latter part of pregnancy is the improvement of vasculopathy-related digital ulceration due to the physiological vasodilation associated with pregnancy, potentially alleviating Raynaud’s symptoms. However, Iloprost infusions may still be necessary, particularly during winter. Dyspepsia secondary to oesophageal dysmotility may also worsen.

Delivery planning is as critical as pregnancy planning. Although there are no contraindications to a vaginal delivery, these patients have a higher risk of requiring Caesarean section. They should be well-informed of the benefits and risks of both methods.

The psychological impact on the family should not be forgotten. Opting for a high-risk pregnancy is very brave, knowing it endangers both the mother’s and baby’s health and that the mother may not survive long enough to see her child grow. This situation can place immense stress on the family and emotional and psychological support is essential.

High-risk patients necessitate close monitoring in a tertiary centre, with regular blood tests, foetal scans, echocardiograms and a clear birth plan.

**Key learning points:**

• Pre-conception counselling and family planning are crucial for cardiac transplant and rheumatology patients due to the teratogenic effects of some immunosuppressive medications and the need to minimize maternal and foetal risks. Pharmacological counselling is vital. Awareness of medications to stop upon pregnancy confirmation is necessary. Close monitoring of drug levels ensures the underlying medical conditions remain in remission, with medication doses adjusted, especially in cases of hyperemesis gravidarum.

• The risk of transplant rejection is lower if pregnancy is postponed until at least one year post-transplantation, ensuring graft function stability. During this initial period, patients are typically on intense immunosuppression. Human leukocyte antigen (HLA) testing should be performed as incompatibility between the donor, father and mother can increase rejection risk.

• Close cardiac monitoring is required, including regular echocardiograms to ensure good graft function. Physiological changes in pregnancy, such as increased cardiac output and circulatory volume are generally well tolerated if this is the case.

• Maternal complications include miscarriage, hypertension, pre-eclampsia and infection. Regular monitoring throughout pregnancy is required including frequent blood tests. Immunocompromised patients should be closely monitored for infections as they can be asymptomatic or present atypically.

• Foetal health is closely tied to maternal health. Complications such as growth restriction and preterm delivery can occur if maternal hypertension or pre-eclampsia occurs. Calcineurin inhibitors like Tacrolimus increase the risk of hypertension.

• Different delivery options are available though there is a higher risk of caesarean section. Delivery should be planned where specialist cardiology transplant services are available. Patients should be monitored for arrhythmia and graft dysfunction due to postpartum volume shifts, although these changes are usually well tolerated.

• Multidisciplinary team involvement, including obstetrics, cardiology, rheumatology and neonatology, is essential for counselling high-risk patients about pregnancy. This patient was immunocompromised due to severe systemic sclerosis and cardiac transplantation, necessitating comprehensive and coordinated care.

